# Eosinophilic esophagitis in Portuguese children: clinical and allergological characterisation

**DOI:** 10.1186/2045-7022-5-S3-P44

**Published:** 2015-03-30

**Authors:** João Marcelino, Rita Aguiar, Fátima Duarte, Ana Célia Costa, Manuel Pereira-Barbosa

**Affiliations:** 1 Immuneallergology Department, Hospital de Santa Maria, CHLN, Lisbon, Portugal

## Introduction

Eosinophilic esophagitis (EoE) is a clinicopathologic entity defined by symptoms of esophageal dysfunction and histological eosinophil (eos) inflammation. Its patients (pts) commonly present concurrent reactivity to both food and aeroallergens, outlining the importance of an allergological evaluation.

## Objectives

Clinical and sensitization profile description of the pediatric population with EoE diagnosis (EoEd) in our Immunoallergology Department.

## Methods

Retrospective study using the EoE database (Feb2009-Mar2014) of pts up to 18 years (yrs). Outcomes were characterization of demographic, symptomatic, laboratorial (peripheral eos, total and specific IgE), endoscopic and histological features and sensitization profile (prick and patch tests).

## Results

We included 24 pts [(21M,3F;10.6(4-16)yrs)]; median age at EoEd: 7(1-14)yrs. Average between symptom onset and EoEd:19±30 months. Average follow-up for 28 (5-60) months.

Most frequent symptoms (%): dysphagia (54), impaction (50), GERD-like symptoms (42), abdominal and/or epigastric pain (42), vomiting (33). Most frequent onset symptom: dysphagia (46%). 96% of pts had prior history of atopy (p<0.001). Average peripheral Eos count 627cel/mL (170-1830) and total IgE 524kU/L (25-2798). At EoEd, the most common endoscopic finding were furrows(79.2%). Histologically 29% had>40eos/High Power Field and 33% had microabcesses (which were more frequent in patients with impactation [p<0.1]).

After EoEd, 96% had confirmed allergy with 92% to aeroallergens (p<0.01) and 75% to food (P=0.023).The main allergens were(%): food [shelfish(61), milk(44), cereals(39), egg(39), meat(33), nuts(33), fish(22), fresh fruits(11)]; mites (75); pollens (54.2).

After a 12 month therapy, all had symptomatic improvement; with 8.3% achieving biopsy normalization.

Of those with pre-EoEd allergenic testing, all had acquisition of new sensitizations (food and/or aeroallergens) after EoEd.

## Conclusions

The first and the most frequent symptom was dysphagia. The prevalence of allergic sensitization was >95%. The potential clinical severity of EoE justifies the multidisciplinary evaluation for clinical, diagnostic and therapeutic workup.

